# Women and the coloniality of urban atmospheres of terror in Rio de Janeiro’s favelas

**DOI:** 10.1177/02637758251334335

**Published:** 2025-05-15

**Authors:** Anne-Marie Veillette

**Affiliations:** University of Pennsylvania, USA

**Keywords:** Affective atmosphere, terror, women, coloniality, favelas, urban borders, body, Brazil

## Abstract

This essay examines the urban atmospheres of terror in the favelas of Rio de Janeiro, Brazil, from the perspective of women residents. Drawing on two ethnographic projects conducted in various favelas in 2016 and 2019, I argue that terror, as an urban atmosphere, is deeply rooted in a long history of racialized and gendered violence, and that its persistence in the contemporary urban landscape is a consequence of the coloniality of power. The analysis begins by exploring the layers, textures, and complexities of urban atmospheres of terror, providing a deeper understanding of their racialized and gendered nature. It further examines the transformative power of the body in reshaping these urban atmospheres, focusing on how favela women cultivate alternative affective atmospheres within their communities. Drawing on Afrodiasporic and decolonial feminist thinking, I show how Afrodescendant women in the favelas resist and transform these atmospheres, creating spaces that challenge the coloniality of power and its spatial manifestations, such as urban borders. I conclude that a key aspect of favela women's urban politics and resistance to coloniality is rooted in the body and the affective dimensions of urban life.

## Introduction

One late Friday afternoon, I exit the metro station Engenheiro Rubens Paiva, a metro station situated in the far north zone of the city of Rio de Janeiro, Brazil, in the neighborhood of Costa Barros, where an important complex of favelas is situated, named Complexo da Pedreira, as well as Lagartixa, the favela where I am going. As usual, I head directly to the moto-taxi waiting area, where only two motorcycles are available. The drivers hesitate to take me, as none of the other drivers have returned from their previous trips. One of them jokingly remarks that I am going “right into the middle of a war” (*você vai no meio da guerra*), but he agrees to take me anyway. As we set off, I become nervous. This is not my first time taking the route following the Complexo da Pedreira, nor is it the first time I see armed police along this road. However, this time, they are visibly prepared for action, and their heightened agitation is palpable. My hands clench, and I freeze on the small seat of the motorcycle. The driver's joke runs in my mind, and I can’t shake the tension off. Despite the roar of the accelerating engine, I ask the driver not to take any risks, telling him I would rather return to the subway station if necessary. He responds with a nonchalant gesture and veers off the main road, where police have already set up barricades. Instead of continuing on the usual path, he takes a side street, where we immediately encounter the traffickers—also seemingly prepared for confrontation. We press on and reach my destination, Juliana's house, a few minutes later, shaken by the experience.

At Juliana's house, she and two of her friends are having lunch. The atmosphere in the kitchen is light and jovial as they chat. When I share what just happened, one of them decides to leave right away. She lives on the other side of the road and fears being stuck here, unable to pick up her son from school. We spend the afternoon at Juliana's place. We remain in the kitchen, a safer space away from the potential danger of stray bullets, and wait for the situation to settle before going out again. By the end of the afternoon, as the sun begins to set over the hills, we venture outside. Fewer vans (small buses serving as public transport) are running, but when one finally arrives, the driver informs us that a police officer has been killed during the confrontation. The commotion on the main road blocks most vans from passing. Nonetheless, the streets we pass through slowly begin to revive, with people returning to their normal activities and windows and shops gradually reopening.

I distinctly felt like my moto-taxi ride brought me into a temporal vortex, passing from a normal afternoon in the city (in time of peace) to a war zone (in time of war) in the blink of an eye. Although I had lived through other police operations and confrontations in the favelas, I had never before “transited” through it. On that day, the fear I experienced resulted not only from the high tension I witnessed and felt between the police officers and the drug traffickers, but also from the sudden change that occurred while circulating in and out of the “conflict zone.” Simply put, I had the distinct impression of entering a vortex, in which time and space were suddenly “other.” In other words, I had crossed a border, that was both spatially and temporally defined, but also affectively, crossing into an atmosphere of terror.

Having conducted various research projects alongside women in the favelas of Rio de Janeiro, I first aim, in this essay, to expand the concept of urban atmosphere of terror to better understand its inner working in ordinary times of war, in line with Sara Fregonese's (2021) effort to deconstruct the dichotomy between cities “at peace” and “at war.” For this reason, this paper focuses specifically on Rio de Janeiro, Brazil, who has a long historic of urban violence. Brazil has not “officially” been at war on its own territory—or close to its own territory—since the War of the Triple Alliance in 1864–1870, nor has it known what the Global North refers to as “terrorist attacks.” However, Brazil's long history of internal pacification and continued political violence against the marginalized segments of its population has often led to militarization and war-like operations (Wacquant, 2008; Zaccone, 2015), including in the case of Rio's favelas (Leite, 2012). As such, although we could identify the specific temporality of the “war on drugs” conducted in the favelas (roughly from the last decade of the twentieth century), the continued “violence” perpetuated at various scales against and within the marginalized sectors of the Brazilian population rather points to the broader temporal horizon of coloniality.

Building on our interconnected experiences in the favelas—myself, a White foreign woman “entering the favelas,” as illustrated in this introductory ethnographic vignette, and the residents, most of whom have spent the majority of their lives there—I aim to unpack the colonial dynamics underlying the situated ways affective atmospheres are experienced. I seek to further explore the gendered nature of urban atmospheres. Drawing on the critical perspectives of Afrodescendant and Latin American feminists, I conceptualize gender as an inseparable component of the coloniality of power, intersecting with race and class (Akotirene, 2019; Lugones, 2010). Drawing from empirical data collected in two distinct projects conducted in the favelas of Rio de Janeiro in 2016 and 2019, I first argue that the urban affective atmospheres of terror in the favelas are deeply rooted in a long history of racialized and gendered violence, tracing back to the colonial plantations and continuing into the present-day urban landscape. Second, I contend that one critical, yet often overlooked, means by which women envision and transform their urban environment is through their intervention in the affective dimension of urban life. Specifically, women from the favelas challenge the pervasive experiences of war and terror by actively (re)creating spaces and moments of peacefulness within the city. In doing so, they shift, disrupt, and potentially destroy the urban borders (the vortex spoken of above) that profoundly shape their experience of the city as a whole.

## Contextualizing fear and terror in Rio de Janeiro

In Latin America, over 80% of the population lives in cities, making this region one of the most urbanized on the planet. However, the growth of cities, especially larger ones, has been strongly associated with a “loss of control” by relatively new democracies in the face of globalized urbanization processes, one expression of which is the growth and proliferation of urban areas built, occupied or inhabited by low-income, racialized or marginalized segments of the population.

In Brazil, and more specifically in Rio de Janeiro, these areas, known as favelas, have been a central urban concern for politicians, urban planners, and residents throughout the city's modern history, as they have almost always been perceived as threats, related, in turn, to hygienist, aesthetic, political, economic, security, and environmental concerns (Gonçalves, 2010; Valladares, 2005). In response to this fear, favelas have been the subject of several targeted urban planning interventions, in order to secure (with military-police operations), relocate, revitalize, or integrate them, often to the detriment of those displaced toward the peripheries by such initiatives (Albert, 2022) or caught between police, militias, and traffickers (Alves and Evanson, 2011; Misse, 2008; Silva, 2015). Even though the perceived source of the threat has changed over the decades, the emotion associated with favelas has remained the same and is firmly rooted in fear (Zaluar, 2006), following the city's affective economy. As argued by Sara Ahmed (2004), fear is the fruit of the circulation of emotions and affects between bodies that “produce the surfaces and contours that allow all kinds of objects to be delimited,” including urban space. This affective economy is embedded in social, political, and historical trajectories that direct the circulation of emotions like fear, which come to “stick to” bodies and spaces—especially those perceived as “foreign” or “other.” Thus, like many works in feminist geography have pointed out, “fear” is not simply an instinctive response (written into human biology) to a “threat” (real or perceived), but the fruit of a process of social othering, which is rooted in social relations and the production of space (Bondi, 2007; Mehta and Bondi, 2010; Pain, 2009; Sandercock, 2016 [2005]). As a result, fear draws urban boundaries that affectively *and* racially delimit certain spaces, such as favelas, from the rest of the city. Or, in other words, fear operates a form of spatial and racial containment of the dehumanized “other.”

Many favelas are the stage of violent confrontations between armed actors, including the police, who have been engaged in an open war against drug trafficking. These police operations, which represent but one aspect of violent pluralism (Goldstein and Arias, 2010; Veillette, 2020), have contributed to producing an urban atmosphere of terror in many of the city and the region's favelas, some of which have punctually or regularly known open armed conflict. In these cases, the local population often feels trapped in a never-ending war, being unable or unwilling to leave their neighborhood. For the police officers, for whom these favelas become their everyday battleground, these neighborhoods have become strongly perceived as battlefields and affectively associated with fear, suffering, and passion. As such, as Paul Pauschinger (2020: 512) has argued, “emotions contribute to police territoriality” in a way that is “shaped by and shape[s] the city as a ‘bordered space.’”

Several studies have documented the effects of fear on urban transformation, notably in São Paulo, where the elite retreated in auto-segregated fortified enclaves while supporting brutal forms of containment and punishment toward the poor, marginalized, and racialized portion of the population (Caldeira, 2000). Thus, while fear has established itself as an urban atmosphere (Gandy, 2017) in many Latin American cities (Pedrazzini and Desrosiers-Lauzon, 2011), its intensity varies greatly depending on one's social position (according to gender, race, and class) and one's spatiality within the city (their neighborhood, localization, land-use rights, and movements) (Leff, 2021; Wetherell, 2013). Furthermore, fear co-exists with a myriad of other emotions, including terror.

In marginalized and peripheral urban areas of Brazil, for instance, many studies show that necropolitics, states of exception and politics of urbicide (re)produce forms of terror rooted in anti-Black violence (Alves, 2018; Smith, 2016; Vargas, 2013). Reminiscent of the plantation regime, the experience of terror and fear racially delimits—or divides—urban space (McKittrick, 2011). For women living within these neighborhoods and urban areas, the experience of fear and terror is further mediated by the reproduction of “boundaries” (socially and spatially defined) upon which the exercise and reproduction of gendered violence depend (Wilding, 2012).

## Expanding on urban atmospheres of terror

The affective dimensions of these urban transformations, however, are not limited to the delimitation of distinct urban spaces. These urban spaces also have an affective charge of their own, or what Ben Anderson (2009) calls affective atmospheres, which could commonly be referred to as collective moods or social climates. Anderson (2009: 80) briefly describes the concept of affective atmosphere as “a kind of indeterminate affective excess through which intensive space–times can be created.” Sometimes, certain affective atmospheres envelop entire cities (e.g., a generalized sense of insecurity). However, the intensity and incessant flow of interactions and encounters between bodies in urban space suggests instead that they constantly emerge and disappear, superimposing on one another (Gandy, 2017). While the concept of affective economy allows us to study how fear delimits favelas from a structural point of view, that of affective atmosphere allows us to apprehend how this fear is experienced on both sides of urban borders. The urban atmosphere of fear is thus traversed by the inexorable paradox between generic categories of experience linked to the city and distinct urban atmospheres associated with specific spaces and bodies.

The current literature on urban atmospheres of terror mainly deals with cases of cities in the context of declared armed conflict (Fregonese, 2017; Pasquetti, 2019), post-conflict (Laketa, 2016), or in the aftermath of terrorist attacks, in the case of cities situated in the Global North (Fregonese and Laketa, 2022; Molotch and McClain, 2003). But in Brazil, and in Rio de Janeiro, the extended temporality of the “war” on marginalized, peripheral, and Afrodescendant communities, heavily contribute in the production and reproduction of an affective atmosphere of terror. Thus, on the one hand, our understanding of the (re)production of urban atmospheres of terror and their longer-term effects on urbanization processes within the Global South, and more specifically Latin America, remains limited. In addition, at the scales of the city and of the community, this war includes a variety of ordinary actors, who are, at the same time, allies and enemies, creating an emotional confusion which I will refer to as “a war raging in the body.” On the other hand, although many contributions within the literature on affective atmospheres embrace some feminist key interventions in geography—articulating emotions and rationality rather than conceptually opposing them, to give but one example among many—few have empirically studied and included the gendered dimension of affective atmospheres, usually focusing on specific categories such as “urban margins” (Pasquetti, 2019) or ethnicity (Laketa, 2016).

If the affective economy of these urban atmospheres shapes the surface of bodies and spaces, it is also because they are anchored there, and so subjects can intervene to redirect, deviate, transgress, change, even replace or eliminate them altogether. In this essay, I take up the idea that affects are defined as “bodily capacities to affect and to be affected that emerge and develop in concert” (Anderson, 2014: 9). From a feminist perspective, one major point of contention with Anderson's concept of affective atmosphere, and somewhat with affect theory in general, has been its treatment of the body as an empty entity, channeling affects of which it is unaware (cognitively, at least) and on which, in the end, it has little power (Leff, 2021). Because affects are generally conceptualized as noncognitive and thus out of reach of any conscious effort, they appear to be disconnected from human (and sometimes nonhuman) agency. For this reason, Margaret Wetherell (2013) has notably argued in favor of noticing the affective practices through which recognizable repetitions indicate that actors are deliberately taking action. As such, these repetitions exemplify one way through which affective atmospheres can be deliberately created and produced as a form of micro-political hopeful promise (Leff, 2021: 5).

Far from arguing that favelas represent monolithic spaces that can either be safe or unsafe, my aim is rather to put forth the fact that urban borders are constantly (re)produced through everyday practices and, in particular, by the very “mundane” act of seeking and creating peaceful spaces for oneself and one's loved ones. As Leff's (2021: 6) intervention suggests, “affective atmospheres take advantage of the circulatory nature of atmospheres and the porosity of bodily borders to move fluidly from the local to the global, the personal and the geopolitical.” Articulating this reflection with Ashon T. Crawley's (2017) on breathing, he continues: “we can recognize the circulation of affect as having an important orientating effect on the world. One that […] shapes our interactions with the world as it is and conditions the possibility for cultivating affective atmospheres capable of making otherwise possible worlds” (Leff, 2021: 6).

Accordingly, the “urban atmosphere of terror” examined throughout these pages is not conceptualized as “something *in* relation between people, place, and things,” but as “atmospheric practices” that “unfold *as* the relation” (Bille and Simonsen, 2019; Simonsen, 2007), with the relation being that of coloniality. Thus, I am precisely concerned with women's practices within the affective dimension of urban life, as they have the potential to generate other affective atmosphere than terror, and, in doing so, create, orient, shape, or transform associated processes of urbanization.

I define “a process of urbanization” as any phenomenon that induces, shapes, or changes the configurations, meanings, or representations of urban space, the latter being understood as “a tangled web of agreements, maneuvers and antagonisms between changing constellations of materials and bodies” (Simone, 2021: 1341). This broad definition makes it possible to integrate several scales and temporalities of analysis, including those of the body as an “urban infrastructure” insofar as it represents a fundamental support for urban life and urbanization processes, particularly their affective dimension (Simone, 2004; Truelove and Ruszczyk, 2022).

## The coloniality of terror

Reflecting on terror in the favelas is necessarily to reflect upon the coloniality of emotions. As suggested by Diana Marcela Gómez Correal (2019: 83) in the case of Colombia, terror is not only an effect of the prolonged experience of socio-political violence, but a tool in the perpetuation of coloniality, working “as a global logic of dehumanization that is able to exist even with the absence of a formal colony (Maldonado-Torres, 2018: 36).” To be—or to feel terrorized—is to be and feel “wretched” in the sense compelled by Franz Fanon (1963): it is to be imprisoned outside the boundaries of humanity (McKittrick and Woods, 2007: 74). Within coloniality, these boundaries are central to capitalist accumulation and always racially defined (Bledsoe and Wright, 2018; McKittrick, 2011). As such, beyond the experience of violence and its effects, an important feature of “terror” is the feeling of spatio-temporal imprisonment, within a system that pretends to and produces a universalized vision—or a totality—without “escape” or “exteriority,” that is, coloniality (Mignolo and Walsh, 2018: 197).

As defined by Walter D. Mignolo (2011: 171–172), in line with other well-known decolonial thinkers, coloniality “is a colonial matrix of power through which world order has been created and managed.” Both the creation and management of this coloniality depends on both temporal and spatial bordering. For instance, in Eurocentric History, Africans only temporally “enter” modernity *when* they are conquered, enslaved, displaced, or eliminated. Their very condition of temporal “appearance” within the realm of coloniality/modernity lies in the sur-imposed experience of terror. References to “plantation memories” to address “episodes of everyday racism” (Kilomba, 2019) or to the “wake” of slaves ships to think about Blackness (Sharpe, 2016) are, on this matter, very evocative. They convey this sense of continued temporal “entrapment” in a matrix of domination that has no beginning (coloniality as the beginning of History) nor end (no alternative futures to coloniality). Terror, as such, is the sur-imposed “emotional habitus”^1^ of the “racialized” within the temporality of coloniality/modernity.

Within coloniality, terror is also spatially defined. Referring to the “affective dimension of urbanization processes,” this essay is in line with the geographies of emotions and affects, for which emotions, affects, and feelings constitute a fundamental part of social agency and the production of space, at various scales of analysis (Bondi, 2007; Pain, 2009). Although these two strands of literature tend to conceptually separate emotions from affects (Pile, 2010) and thus argue that their objects of study (i.e., emotions for one and affects for the other) differ, I choose here, like a growing number of geographers and urbanologists (Ansaloni and Tedeschi, 2016; Barclay and Riddle, 2021; Davidson and Milligan, 2004; Laketa, 2016; Leff, 2021; Mehta and Bondi, 2010; Prouse, 2021; Simonsen, 2007; Wetherell, 2012), to treat them as inextricable arrangements from the point of view of experience.

## Accessing the affective dimension of urbanization processes

To access this affective dimension, the body is a fundamental key (Ahmed, 2004; Ahmed and Stacey, 2001; Anderson, 2009, 2014; Leff, 2021; Simonsen, 2007). Thus, following Kristen Simonsen's (2007) suggestion to adopt a “social ontology of practice,” the researcher's body is considered here in its dual role as “object of study” and methodological tool in the sense that, to apprehend emotions and affects, it must be receptive, open, and attentive to the sensations experienced during (and beyond) the research fieldwork (Veillette, 2023). Accordingly, I argue that to *feel* one's way through urban space is necessary to affect and be affected by it, to *feel* when and where we cross borders and, notably, changes in affective atmospheres. The body—as a space and a situation—moves and, in doing so, affectively and sensorially measures the world. In other words, urban borders are felt as bodies move and interact (with each other, nonhumans and infrastructures), picking up the textures, intensities, and layers of fear, producing a visceral geography (Sweet and Ortiz Escalante, 2015) of the city. As I suggest throughout these pages, this visceral geography of everyday life in the city reveals a complex and nuanced portrait of segregation, which could not be perceived or apprehended without an embodied approach to urban and geographical research.

I align with Kathleen's Stewart (2007: 3) understanding of ordinary affects, which “work not through ‘meanings’ per se, but rather in the way that they pick up density and texture as they move through bodies, dreams, dramas, and social worldings of all kinds. Their significance lies in the intensities they build and in what thoughts and feelings they make possible.” In other words, the affective dimension of urban life overflows what we might see or say, and remains something that we feel: like impressions and pressures upon our skin (Ahmed, 2004; Ahmed and Stacey, 2001), but also in the flesh (Simonsen, 2007: 172), and the gut (Sweet and Ortiz Escalante, 2015).

Understanding the body as inherently porous entity (Gómez Correal, 2019: 84), shaped and affected both by our encounters with others, in the same way that we shape and affect other bodies through these encounters, what form of knowledge emerges from this work is profoundly interrelational, in the sense that it foremost streams from the relationship I have built with some of these women since the last 10 years: our shared experiences of fear, terror, anxiety, sadness, and anger, but also, and I have to say mostly, moments of joy, of excitement, of hope, of peacefulness, and friendship (Veillette, 2024). The evidence presented here is not only the result of my own positionality, but the “result” of our relationship, thought of as an ongoing, embodied “practice” and, always in “rehearsal” (Gilmore, 2024) with all its mutual benefits and challenges.

More precisely, the results are based on my last two research projects conducted in the favelas of Rio de Janeiro. The fieldwork of the first research project occurred between May and September 2016 and focused on women's experiences of police violence in the favelas. The fieldwork for the second research project occurred from May to June 2018 as well as from July to December 2019. This second project investigated urban transformations in Rio de Janeiro from the favela women's perspectives and praxes. They both adopted an ethnographic method, which included participant observations (in different activist groups and the participants’ everyday lives), interviews (open-ended and semi-structured), and focus groups. Twenty-one women participated in the first research project, while 60 participated in the second. More precisely, women were—in both cases—recruited through a snowball sampling method. To represent the diversity of urban realities in Rio de Janeiro, the women were recruited from all areas of the city, of all ages (with the exception of minors), sexual orientations, abilities, religious beliefs, and political ideologies.

From a feminist standpoint, this embodied method allows me to integrate three essential elements to the methodology. First, it takes into consideration the fact that not everyone—including myself—enters affective atmospheres from the same departure points (Leff, 2021), meaning that their formation is directly linked to interrelational, intersubjective, and intercorporeal encounters (at a certain time and a certain place) of differential and differentiated bodies—whether as individuals or collectivities. Second, this methodology embraces a definition of affects as fundamentally intercorporeal and intersubjective, two terms that, put together, can be called interrelational (Gómez Correal, 2019). This understanding of affects means that “affects” and “emotions” can never merely be considered as “objects” of research, nor could they be fully addressed simply by a reflexive engagement of the researcher with them. Because they form an “affective economy,” the feelings, affects, and emotions translated into these pages emerge from shared lived experience, located, as Simonsen (2007: 171) would put it, “in the ‘mid-point’ between mind and body.” Third, in accordance with Jack R. Leff’s (2021: 2) argument, this approach seeks to leave “meaningful room for political agency.” As such, affective atmospheres are social phenomena, which means they do not merely use the body as a passive support or tool. Bodies orient their circulation and can thus, as I argue in this paper, contribute to transforming the affective landscape of the city.

## The layers and textures of urban atmosphere of terror in the favelas

### The war raging “on” the body

The spatiotemporal experience of violence was a recurring theme during the interviews and focus groups conducted in the favelas, given, not only my interest in the matter but also the everyday reality of many of the favelas I came to know as a researcher. The experience of terror, however, was generally expressed as a feeling of entrapment in more or less delimitated space—like the favela—and time—especially a time of war. One of the women met in Lagartixa, spoke directly of this during a focus group I conducted at Juliana's house:

I think that there's literally already a third war here in Brazil. The war has become so established here that we're just hostages. There are war refugees, but we're at a level where we have nowhere to go for refuge, so it's like we're prisoners of war, prisoners within our own space. (…) If I leave here, go to a neighboring neighborhood, go to another one, or another one, I'm going to experience the same violence, in different ways, but it's also going to be violence, so we don't have much of a place to go, so I think the city of Rio de Janeiro (…) has already surrendered, so, in my fatalistic conception, I think there's no more improvement, this is it, it's done, are you going to live (*conviver*) with it? (Focus group, Lagartixa, 2019)

The entrapment described in the intervention of this young mother reflects two critical dimensions of the pervasive atmosphere of terror she experiences. First, there is a sense of temporal stagnation, in which the individual perceives time as cyclical, leading to the belief that history is condemned to repeat itself. As expressed, “I think the city of Rio de Janeiro (…) has already surrendered, so, in my fatalistic conception, I think there's no more improvement.” This indicates a fatalistic outlook in which any potential for positive change seems increasingly unlikely. Second, there is an awareness that the terror they endure transcends spatial boundaries, extending beyond their immediate environment. Despite physical relocation, the individuals involved are acutely conscious of the territorialized nature of the violence in their community and the possibility—if not inevitability—that this terror will follow them. Belonging to a specific socio-political and geographical context—the favelas—shapes their experience of terror. This sense of entrapment, defined by race, class, and territory, is inescapable. Even if they leave, they remain tethered to the favelas, which function like a gravitational force that continually pulls them back. Here lies one major difference between my own experience of fear while visiting a favela as an outsider, and the terror experienced by the residents.

The atmosphere of terror further upon a third critical dimension: uncertainty. What my body responds to, as I notice police officers and drug traffickers retreating behind made-up barricades to start shooting, is what residents usually refer to as a “tense climate” in the favela. However, as Menezes et al. (2024: 4) have argued, cohabitation with violence in many favelas involves the capacity of the residents to read the climate “no only through a rational calculation, but also through an ‘ecology of the sensible’ in the favela.” Accordingly, “this way of feeling is largely based on the ability to identify “clues” in the environment that make it possible to measure the “climate.” This becomes an especially valuable “skill” when evolving in an environment where the shift between tension and explosion is hardly predictable.

Everyday life in tense favelas like the Complexo da Pedreira, where Lagartixa is situated, necessitates learning to distinguish the variations and textures of terror, something that, as an outsider and a foreigner, I had not learned to do. If the residents of favelas like Lagartixa had to stop what they were doing every time the situation was tense, then they would always need to stand still. My motorcycle driver, like Juliana, had learned to distinguish the levels of tension in the atmosphere, to feel it rising, but also to discern the time left before the explosion. Their developed sensitivity allows residents to capture the subtleties and textures of affective atmospheres, even when the overall atmosphere is heavy or tense, as this excerpt from the Lagartixa focus group (Complexo da Pedreira, in the Costa Barros neighborhood, north zone) shows:

Participant: In our case, fear is necessary because it is fear that guarantees our survival, if only to get around, we can feel the climate. It's a bit like when I said earlier, that we look to see if there are police, but nothing is happening… We feel it. We know that the motorcycle cabs are running, that the businesses are open…

Participant: When there's tension, it's different, you know that.

Participant: It gets… different.

Participant: It's like when we came in this morning […] there were people looking towards something. We already know more or less what's going on, without seeing what those people are seeing.

Participant: Everyone looks at the same thing.

Participant: Everyone is looking the same way, people are reacting, action-reaction. And so, when you've lived here your whole life, you end up having a differentiated view of violence that someone from the outside certainly doesn't have. They don't have the tact (*o tato*) to perceive that the situation is changing. (Focus group, Lagartixa, October 25, 2019)

This excerpt speaks, first, of these women's ability to “read bodies”: to conclude that something is happening because people are all looking in the same direction and to react accordingly. It could be argued that favelas’ residents have learned to identify specific clues Menezes et al. (2024), or even what Elijah Anderson (1990, 1999) called the “street code.” In this case, they mention that the stores are closing, the motorcycle cabs are stopping as a sign that something is going on, and everybody seems to be looking in the same direction. However, they also try to describe that something is in the air (“we can feel the climate”), without really being able to put their finger on what it is exactly. There is what they see bodies doing in the space, but there is also something else; something seemingly elusive and more subtle at work (“the tact of perceiving that the situation is changing”). These atmospheres can be felt in the way bodies hold and move, but they can also be felt in the flesh and on the skin, where they leave impressions, much in the way that one walks out of one's home and can feel a storm coming on. But when the storms follow one another, one becomes more sensitive to the subtleties, textures, and layers of complexity that differentiate one storm from another. This sensibility seems to be implied by the focus group participant when she speaks of “tact,” and explains the discrepancy between my own experiences of potentially dangerous situations in the favelas and those of the residents I met. As such, the affective atmospheres of terror in Rio de Janeiro are mediated by the body's exposure to them and accumulated knowledge, that is, or what I call the “war raging *on* our bodies.” When crossing urban borders then, the affective contrast is sometimes so intense that one might temporarily become insensible to these variations. However, with time, the body becomes more sensitive to the textures of urban atmosphere and, thus, alters the subjective ways through which urban borders are being (re)produced and how they are reconfigured, transgressed, and dismantled.

### The war raging “in” the body

Despite the various strategies residents develop to cope with their environment, one of the persistent dimensions of terror in favelas is the unpredictability of violence and the accompanying uncertainty. While favelas are not continuously in a state of tension or conflict, moments of heightened fear and instability often emerge in response to the shifting movements of armed groups within the city, as well as the implementation of urbanization initiatives at various levels of governance. These efforts include, without being limited to, the recent efforts aimed at “pacifying” favelas, through the militarization of their territories by special military police units (Fleury, 2012; Leite et al., 2018; Vargas, 2013; Veillette, 2017). A resident of Complexo de Alemão expresses this sense of unpredictability very clearly during our interview:

It's tolerable when things are calm, but when a confrontation occurs—when there's a conflict—everything changes. A lot happens, many lives are lost, many deaths occur. I don’t want to spend my entire life here, witnessing these events. For a while, it's fine; the favela is peaceful, everything seems calm. But then, a war breaks out, and suddenly five, ten, twenty people are killed—I don’t know. I don’t want to be here, watching this. I don’t like it. (Interview with Denize, resident of the Complexo do Alemão)

Although individuals may adopt various adaptive strategies to navigate and protect themselves from the ever-present threat of violence, these responses do not eliminate the underlying sense of terror. As illustrated in the following interview with a former resident of Cantagalo, a favela located in the wealthy southern zone of Rio de Janeiro, this pervasive fear remains an inescapable part of life in these communities:

For example, you might walk down the street and see the body of a young boy lying there, and you’re forced to ask yourself why. In reality, this is the impact of drug trafficking—they control the community through fear and violence. They kill one person in front of others, causing shock and trauma. The most shocking experience for me was with a sixteen-year-old boy I had known since he was in his mother's womb. I was walking past the market carrying my shopping bags when I saw him. His name was Emerson. I called out, “Emerson, what are you doing here? This is no place for you in the boca de fumo^2^.” He looked at me and said, “Get me a job, and I’ll get out of here.” A sixteen-year-old boy, asking for a job so he could leave the drug trade. Half an hour later, as I was walking back, I heard gunshots. The news quickly spread that Emerson had been killed by the police. I had been near him just twenty minutes before. It was just one of many examples. This happens regularly—one day you’re talking to a boy, and the next, he's dead. And it's not just one or two cases, but many. These are boys I saw grow up, and now they’re dead, either at the hands of the police or of the drug traffickers. All of this shook me deeply. As I neared fifty years old, I realized I couldn’t continue living like this. I wanted some peace. It was too much. That was one of the key reasons I decided to leave the community. (Interview with Claúdia, ex-resident of Cantagalo)

In contrast to the warring state created both by police and drug traffickers in many favelas—and the increasing power of militias in many of them, if not most (Araújo, 2024; Benmergui and Soares Gonçalves, 2019; Hutta, 2022; Zaluar and Conceição, 2007)—seeking spaces and moments of peace was invariably mentioned by the women I have met in the favelas in Rio de Janeiro through the years.

This yearning for peace (on which I elaborate further in the next section), however, was heavily mediated by their gendered experience of terror, marked not only by armed violence and conflicts within their community (Veillette, 2020; Veillette and Nunes, 2017), but also by the one experienced in intimate relationships (Hume and Wilding, 2020; Krenzinger et al., 2021; Moura, 2008). Many years after our interview in 2019, Denize passed away after having suffered two heart failures in a few days. During our interview, I had asked Denize about her strategies to protect herself from violence, and she made it clear, as the majority of the women who participated in this research project, that most of these strategies were aimed at the partner or the husband, or in other words, to the man she shared her life with. She admitted having had to repeatedly and violently defend herself from her husband in the past. Like many other mothers of young Afrodescendant young men in the favelas, she was also increasingly worried about her son, who was being pulled into trafficking at the time. As per common knowledge in the community, I was made aware that her son had been imprisoned at some point after 2019. Although her passing away was not directly due to direct violence (or at least, not that I would know of), it is undeniable that repeated experiences of terror, whether they are related to armed conflicts or intimate relationships, take a toll on women's health and global well-being, and sometimes a fatal one. As generations of feminists have argued, the home is often far from a safe place for women, and this proves to be especially true in the favelas where the various legal protections that women can seek (at least, in principle), are often less accessible, applicable and well-implemented than in other neighborhoods. Denize was 39 years old when I interviewed her in 2019, and her passing away exemplifies the deep consequences of the repeated stress, and trauma inflicted by terror and its various layers. Far from a sad occurrence, Denize's faith is connected to many other Afrodescendant women across the favelas and other urban marginalized communities across Brazil,^3^ whose everyday experience of terror presses upon their skin, and gradually sinks into the body. As such, the concept of atmosphere of terror allow us to better understand how coloniality works from within the favela, but also the body, killing not only with guns and neglect, but also from the long-term impacts of violence and terror. The atmosphere of terror in the favelas is characterized by feelings of temporal stagnation, entrapment, and uncertainty. But it is by picking up its textures and layers that we can grasp the myriad and intricate ways through which coloniality is continuously being played out in everyday life, and most importantly for our argument here, in our body. The atmosphere of terror—with its complexity and contradictions—feeds “a raging war” within the body, which Fernanda, a resident of the Complexo do Alemão, describes very acutely during our interview:

[Violence is] when you want to lie down to sleep, but you hear people screaming for help, and you can’t do anything to help them—it tears your heart apart. I remember a time when I was [working at this health institution^4^, close by]. I would tell the girls there what had happened—this, that, and many other stuff. And it felt like I was in a completely different world, a world that wasn’t the one I was actually living in. It's the inability to help people who are suffering that sometime feels worse than harming your own body. That's violence, too. But then there's the violated violating, because in reality, those people from the favela who are labeled as “bandits”—I don’t call anyone a bandit—are the ones who really suffer. For me, a bandit is someone who holds the people's money, someone who should be using it for the good of the people but instead uses it for their own benefit, for their family. That's a bandit—that's the miserable, the wretched. I don’t even know what to call them. You don’t know the magnitude of it… It's not about revolt; I’m not angry. I’m just sad to be part of a society that treats Black people, favela residents, [and] the poor, who have nothing, [this way] … while the rich are those with everything. (Interview with Fernanda, resident of the Complexo do Alemão)

Following Fernanda's intervention, this “raging war” within the body can further be understood as a manifestation of the conflicting affective experiences that arise from a violent form of sociability (Machado da Silva and Menezes, 2019), which permeates interpersonal relationships and, by extension, various social spaces simultaneously (Pearce, 2006). Like other residents of the favelas, women must navigate not only the armed confrontations that periodically erupt in public spaces, but also the complex and often contradictory interactions with the very same violent actors—whether drug traffickers, police officers, militia members, or ordinary men and women—with whom they may form intimate, familial, or communal bonds, such as those with partners, children, brothers, sisters, fathers, mothers, friends, and neighbors. This dynamic adds an additional layer to the affective experiences of women in the favelas—a majority of whom are also racialized and economically disadvantaged—and underscores how such suffering, rooted in these intersecting social conditions, not only manifest within the body but may also create conditions for its ongoing reproduction.^5^

## Nurturing other affective atmospheres in the favelas

### Peacefulness as a sanctuary from war

Throughout the time I have spent in the favelas, I have felt peaceful and safe in a variety of spatiotemporal settings. The first was in the natural parks or community gardens, many of which are actively protected and cared for by women. In Laboriaux, one of the many neighborhoods of Rocinha (an imposing favela situated in the wealthy south zone of the city), an old fountain has long provided the community with clean natural water from the Tijuca National Park. Although today most residences in Rocinha are connected (in one way or another) to running water, many Laboriaux residents continue visiting the fountain daily. Laboriaux is composed of a narrow street and adjacent houses set on the mountain's ridge, giving its residents one of the most impressive views of the city's beauty (the Lagoa de Freitas Lake, the Corcovado, and the faraway ocean). At the end of the road, a path enters the forest, the dense vegetation blocking any view and the busy sounds of one of the biggest favelas in the city. At the end of the path, a small pipe delivers the water cumulated into a pool above the rocky hill.

I headed there for the first time with local friends after a meeting gathering community members and various favela residents actively involved in the struggle against favela removals (*remoções*). The contrast between the heat in the street and the coolness of the forest air was instantaneous. On the path men and women carried old cans overflowing with fresh water accompanied by their children. At the fountains, women activists that also took part in the meeting greeted us happily, telling us proudly that the pool retaining the water above the fountain was a result of their collective efforts (what they refer to as *mutirão*) in the early 2000s. We stayed chatting with them for some time, during which at least two dozen of the local residents came: children played in the water, people bathed, others quickly filled their tanks, while many stayed and exchanged some news with each other. The activists’ eyes were bright as they invited us to notice the peacefulness of the place and the very sacred role the water played in their lives. More than a “commodity” (albeit an essential one), the place offered a sanctuary in a community where fear related to forced removal, policing and drug trafficking sustained a tense atmosphere through the last few years.

I encountered similar feelings of peace at various times in the company of women in the favelas, namely in some of their homes, community spaces (like grassroots nongovernmental organizations or local associations), and religious places of worship (including Catholic and Pentecostal churches as well as Afro-Brazilian terreiros^6^), many of which were created and maintained by women. As such, if affective atmospheres of terror—in all their dimensions, layers, and textures—are an expression and product of the coloniality of power in Rio de Janeiro's urban space, they are neither immutable nor fixed in time. If we agree with Simonsen's (2007: 174) idea that “an important precondition of the production of space is (…) that each living body both is and has its space; it produces itself in space at the same time as it produces that space,” should not this imply that affect has a transformative power? As Jasbir Puar (2017: 19) puts it, “affect is precisely the body's hopeful opening, a speculative opening not wedded to the dialectic of hope and hopelessness but rather a porous affirmation of what could or might be.”

The favela can represent a refuge from a racist city when a domestic worker comes back from an upper-class neighborhood, but it can also become the very place where she experiences the terror of police operations or the loss of a loved one. By “refuge,” I do not refer to Michel Agier's (2015) notion of a shelter from which emerges the “ghetto,” even if, in many ways, this could be said of some of the favelas in Rio de Janeiro. I rather build upon Françoise Vergès's (2020: 17) notion of a “politics of peacefulness (*une politique du paisible*)” which implies not only resisting direct forms of violence—including police violence (Veillette, 2020)—but making sanctuary spaces, where women—especially racialized women—and their loved ones can rest, breathe, and move freely. The acts and attributes of this politics of peace, instead of focusing on how heavy the past and present are, are about imagination and prefigurative acts of (self-)care that bring forth another future, rid of war.

When asked about the type of future they envisioned for both the favela and the city, many participants—including one from a focus group conducted in a *terreiro* of Rocinha—focused not on the visual or structural aspects of that future, but rather on the emotional or experiential dimensions it should embody:

I was reading an article (…) in which one Black boy asked another, what would you do if racism didn't exist? The only answer that really touched me was: “I would run down the street without fear.” Yesterday I was talking to a friend of mine at work and she asked my opinion because her son is Black and he came home from school one day crying a lot, saying: “Mom, I'm afraid of being Black.” So unfortunately today I still know that what I want for this favela, this place where I exist and where I resist, is very far away. (Focus group, Rocinha, 2019)

The far-off future she speaks of is, indeed, one in which favela inhabitants are neither terrified nor scared. In the years leading up to the fieldwork conducted in 2019, Rocinha has experienced relative peace. Fewer gunshots have been heard. Drug trafficking is still present in various *becos* (narrow alleyways), but police patrols on the main roads and entrances to this imposing favela remain “respectfully” distant. As such, the fear of shootouts and police operations has diminished for the time being, allowing Rocinha to be relatively at peace—something Bianca, an elder woman also residing in Rocinha, hopes will endure in the long run:

What I want for my future is a good ruler, public health with doctors, nurses and medicines. That is what I want, because if you get sick… today I'm old, tomorrow if you go to the hospital, you'll have nothing… you turn on the television every day and you see this… and for the favela, I want this peace that is now to continue… that we've been living in this peace without war for two years, there's no war between them (the drug traffickers), nor with the police when they come… that it continues the way it is… and in politics, that those in power do something better for us… because in order to live well, we depend on those in power, for you to have a better life, for you to even have the self-esteem to go out… . (Interview with Bianca, Rocinha, 2019)

Bianca's vision of an ideal future is framed by the expectation that time will not repeat itself; that is, the belief that overall living conditions in the community will improve, including access to essential public services. While she expresses hope that this peace will endure, she simultaneously voices concerns about its long-term sustainability. These concerns arise from the uncertainty generated by the pervasive atmosphere of terror, which infiltrates everyday life and exerts a profound impact on both the body and the modes of being. Although Bianca acknowledges that many of these issues fall under the purview of “those in power,” she also recognizes that she—like other residents of Rocinha—must cultivate the “self-esteem to go out,” a necessary step to dissolve her sense of entrapment. This entrapment is not only shaped by the affective atmosphere of terror in the favela but also by the broader affective economy of the city, which creates and reinforces racialized urban borders. Bianca points out to the fact that, the ability to cross—and ultimately dismantle—these borders relies in significant part on the body, and especially on the body as a situation, that is, which maintains “a structural relationship between our projects (freedom) and the world (which includes the body)” (Simonsen, 2007: 173).

### Rehearsal for peace

This project of freedom is always in practice, or being rehearsed, to quote here Ruth Wilson Gilmore (2024), and depends on transforming one's feeling of smallness into an infrastructure of feeling (for instance, self-esteem). What Gilmore refers to as “abolition as rehearsal” is the body putting into practice what it would feel like to be free, emancipated. If not an “end” in itself, Mônica, an Afrodescendant women and politician from Borel (situated on the hill side of the Tijuca National Park and adjacent to the same name neighborhood), asserts that it offers the auto-care necessary to soot—even if only momentarily—the “war raging inside” to survive and fortify oneself for the “war raging out there”:

There's no institution that would take us in. In fact, we end up having to take care of ourselves. And as a Christian, an evangelical, I have faith. So, at those moments, we turn to the transcendent, believing in a higher power—a greater conscience—which we call God, or Jesus, who watches over us and protects us, to some extent, beyond mere chance. That belief helps us maintain faith in people, in some way, and gives us hope for better days ahead. But organizing to fight… it's our own fight, right? My fight, alongside others, against this violent system where the poorest and most vulnerable live, that's also a source of strength. It's collective strength. The struggle itself, facing all of this, becomes a way to… strengthen ourselves emotionally, physically, and stay rational through it all. Not everyone can do that. Unfortunately, not everyone makes it through. It's no surprise that some people lose their minds, commit suicide, or die in more brutal, violent ways. They stop wanting to live, right? They shorten their will to exist because of these violent processes… because of being so close to violence. We end up witnessing very dramatic, very hard scenes. Sometimes it's good that we have defense mechanisms—mental ones—that allow us to suppress those memories, put them somewhere we don’t remember, and only recall them when, for example, we’re talking about it in an interview or writing. Because these are very traumatic situations, very dramatic. They’re… I’d call them traumas. (Interview with Mônica, ex-resident of Borel, 2019)

These practices of freedom, or moments and spaces of rehearsal in practice are something deeply rooted in care, everyday life, and intrinsically corporeal (Simonsen, 2007: 171). As such, they are generally more felt than seen, more noticeable through the skin (Ahmed and Stacey, 2001), the flesh (Simonsen, 2007: 172), and the gut (Sweet and Ortiz Escalante, 2015), than consciously named and identified through time and space.

My visits to the park, the water fountain, Juliana's home, and the churches represented shared moments of peace, amid war. They were breaches in the time and space of war where we would share, each in our own embodiment, what it feels like to be fearless. They represented moments where women around me rehearsed being fearless, and in doing so, re-creating, through practice, the favela yet-to-come, the one they yearn for. These momentary breaches in the atmosphere of terror that so heavily presses upon the skin, and colonize the body, were the living expression of a “politics of peacefulness” where other affective atmospheres (than terror) can be cultivated, cherished, and spread. They represent one example, among many other probable ones, of affective atmospheres being forcefully resisted and transformed in order to create something else—or something new—that undermine the coloniality power and its spatial expression (urban borders) within the city of Rio de Janeiro.

## Conclusion

With the emotional and affective turns in social sciences, the affective dimension of urban transformation has become a central concern for a growing number of geographers, urban planners, and urbanologists. But the tendency to reflect upon affective motivations (such as fear) and the effects of certain urban (un)interventions (such as terror) has fostered the impression that fear and terror are a direct consequence of urban life, especially in the Global South, and especially for women. By addressing the complexity of urban atmospheres, this paper has attempted to undo the linear correlation between “public events of violence” and the consequent production of “urban atmospheres of terror,” through an embodied analysis of favela women's everyday life in Rio de Janeiro. The analysis further highlighted the tensions—the war raging “in” and “on” the body—that arise from the colonial difference and from the unresolvable contradictions intrinsic to ethnography. I argue that these tensions are of great analytical value. For one, their existence is living proof of the sociality of urban atmospheres and, as such, a testimony to the fact that they can be re-oriented, bifurcated, challenged, and transformed. Indeed, favela women's practices of rehearsal enable a politics of peacefulness through which atmospheres of terror are constantly undermined, impacting the overall affective economy of the city. If these politics of peace often operate mainly at a hyper-micro scale—including at the scale of the body—they work cumulatively to redraw the borders that tend to separate the favelas from the city. In this sense, they simultaneously reveal and resist the coloniality of the urban atmosphere of terror in the favelas.

Finally, this paper further sought to show the concrete way atmospheric practices transforms cities—even at ordinary times—in line with recent feminist interventions examining the related dimensions of care and reproductive labor (Tanyildiz et al., 2021). As such, the effects of women's interventions in the affective dimensions broaden our understanding of the nature of political action in cities and urbanization processes. For instance, the temporary sanctuaries nurtured by favela women offer living proof that urban borders can be shifted, transformed, or even lifted “from the bottom-up” by intervening directly in the affective dimension of urban life. Acting to transform affective atmosphere is to challenge an affective economy that reinforces urban segregation and violence in the favelas and is, as such, political. However, to be aware of this politico-affective dimension often requires *feeling* and working through the body.

## Notes

1. Here, Gómez Correal burrows from Deborah Gould's (2010: 3) concept of emotional habitus, which she defines as “the socially constituted, prevailing ways of feeling and emoting, as well as the embodied, axiomatic understandings and norms about feelings and their expression.”

2. *Boca de fumo* is a drug selling point.

3. This episode is not without recalling Keisha-Khan Y. Perry's (2013: 120–121) account of Dona Iraci's death by hearth failure during a police intervention in a small fisher community of another Brazilian city, Salvador da Bahia.

4. The exact institution has been taken out of this citation to insure anonymity.

5. Constrains both in space and scope do not allow me here to address directly the issue of women's response to and use of violence within the favelas. For a broader exploration and analysis of this thematic, see Donna M. Goldstein (2003, chap. 4).

6. A *terreiro* is an Afro-Brazilian religious space or temple.
